# Bioaccessibility and Pharmacokinetics of a Commercial Saffron (*Crocus sativus* L.) Extract

**DOI:** 10.1155/2020/1575730

**Published:** 2020-01-30

**Authors:** Paula Almodóvar, David Briskey, Amanda Rao, Marín Prodanov, Antonio M. Inarejos-García

**Affiliations:** ^1^Research and Development Department, Pharmactive Biotech Products S. L. Parque Científico de Madrid, Madrid, Spain; ^2^School of Human Movement and Nutrition Sciences, The University of Queensland, St Lucia, QLD, Australia; ^3^RDC Global Pty. Ltd., New Farm, QLD, Australia; ^4^Instituto de Investigación en Ciencias de la Alimentación, (CIAL) (CEI, CSIC-UAM), C/ Nicolás Cabrera, 9, 28049 Madrid, Spain

## Abstract

There are few studies about the pharmacokinetics of the low-molecular mass carotenoids crocetin or crocin isomers from saffron (*Crocus sativus* L.). None has been performed with a galenic preparation of a standardised saffron extract. The aim of the present research work was to study the effect of *in vitro* digestion process on the main bioactive components of saffron extract tablets and the corresponding pharmacokinetic parameters in humans. Pharmacokinetics were calculated collecting blood samples every 30 min during the first 3 h and at 24 h after administration of two different concentrations (56 and 84 mg of the saffron extract) to 13 healthy human volunteers. Additionally, an *in vitro* digestion process was performed in order to determine the bioaccessibility of saffron main bioactive compounds. Identification and quantification analysis were performed by HPLC-PAD/MS. Digestion resulted in 40% of bioaccesibility for crocin isomers, whereas, safranal content followed an opposite trend increasing about 2 folds its initial concentration after the digestion process. Crocetin in plasma was detected in a maximum concentration (*C*_max_) in blood between 60 and 90 min after oral consumption with dose-dependent response kinetics, showing that crocin isomers from galenic preparation of saffron extract are rapidly transformed into crocetin. The results showed that this tested galenic form is an efficient way to administer a saffron extract, since the observed crocetin *C*_max_ was similar and more quickly bioavailable than those obtained by other studies with much higher concentrations of crocetin.

## 1. Introduction

Saffron, dried stigmas of *Crocus sativus* L. (Iridaceae), was traditionally used as food spice worldwide to provide colour, taste, and odour in several food recipes. Saffron is characterised by a pool of bright yellow-coloured crocins, water-soluble carotenoids of 20 atoms of carbon [1], a bitter taste attributed to picrocrocin [2, 3], and its subtle aroma, chiefly due to the presence of safranal [4, 5].

Besides the culinary role of saffron, safranal and crocin isomers show bioactive properties with several therapeutics and pharmacological applications [6–8], altogether with other antioxidant compounds such as kaempferol derivatives [9, 10]. By contrast, the interest of picrocrocin is limited since it is poorly absorbed in the gut [11]. Nowadays, the clinical evidences of saffron extracts have been accumulated and has been postulated as a complementary therapy in several medical conditions. In particular, to delay the occurrence of the symptoms and effects of degenerative ocular diseases [8] and to prevent and treat mood disorders and mild-to-severe depression without side-effects, even in people with unremitted depression treated with antidepressant medication [12–16]. In depression, different mechanisms of actions have been proposed to explain the functional properties of saffron such as reuptake inhibitors of monoamines, *N*-methyl-D-aspartate receptor (NMDA) antagonism, and gamma-aminobutyric acid (GABA) agonism [17, 18]. In addition, the antioxidant capacity of saffron may contribute to balance the oxidative stress in depressive patients [19]. The anti-inflammatory, antioxidant, and neuroprotective properties may explain its positive effect on the eye [8, 20].

The published beneficial effects of saffron extracts led to better understand the role of its bioactive compounds, but their bioaccessibility and bioavailability were investigated mostly only in *invitro* experiments, cell models, and animal tests. During *in vitro* digestion of an aqueous saffron extract, a significant reduction of crocin concentration was observed, mainly *trans*-crocins, in a dependent process on the physiological pH, temperature, and time of digestion [21]. In experiments with Caco-2 cells, it was found that picrocrocin and crocin isomers were bioaccesible with different efficacy; picrocrocin was poorly or almost not absorbed by the intestinal cells, whereas crocins showed a high bioavailability in crocetin form after its deglycosylation due to an enzymatic action [11, 22]. The *in vitro* studies results were confirmed in animals. After oral dosing of crocins or crocetin, no crocin but crocetin was measured in the rat and mouse plasma as free, mono-, and diglucuronide conjugates [23, 24]. Most of this information has been evaluated with purified compounds, crude laboratory-extracts with diverse composition, or raw saffron stigmas in food. The *in vitro* digestion studies report that the bioaccessibility of crocins depends on different factors such as the dietary ingredient which are consumed with or its combination with other bioactive compounds [25, 26].

Despite the growing interest in the use of saffron extracts as a functional product in mental health, there are few studies about the pharmacokinetics of its bioactive substances in humans. One of them determined the crocetin appearance in the plasma samples of 4 human volunteers after the intake of a cup of 200 mg saffron infusion [27]. The two others tested the kinetics of crocetin in plasma in volunteers that consumed crocetin purified from plant sources at high concentrations [28, 29]. None of them studied the pharmacokinetics of a pharmacological preparation of a standardised saffron extract. A study is needed for a better understanding of the effectiveness and the relation dose-response, being particularly interesting for setting directions for use, doses, and the development of new products based on this extract. Therefore, the aim of this study was to investigate the *in vitro* bioaccessibility of the main bioactive components of a commercial saffron extract (affron®) during the digestion process and the corresponding pharmacokinetics in humans after oral administration.

## 2. Materials and Methods

### 2.1. Materials and Reagents

The saffron (*Crocus sativus* L.) stigmas were extracted in the factory of Pharmactive Biotech Products S. L. in Madrid (Spain) to produce the commercial saffron extract, branded as affron®, and dosed in tablets as preferred galenic form for the *in vitro* digestion and the clinical trial. Each tablet contained 14 mg of dried affron® standardised to ≥3.5% lepticrosalides®, defined as the content of bioactive compounds including safranal and crocin by HPLC [12, 13], together with the following excipients: microcrystalline cellulose, calcium hydrogen phosphate, povidone, croscarmellose sodium, colloidal anhydrous silica, and magnesium stearate as excipients and coated with hypromellose (HPMC), macrogol (PEG) 8000, and carnauba wax.

Safranal, crocetin, *p*-nitroaniline, and digestive enzymes were purchased from Merck (Darmstadt, Germany). Kaempferol was purchased from Extrasynthese (Genay, France), and *trans*-crocin-4 was purchased from Phytolab (Vestenbergsgreuth, Germany). All the solvents used for the chromatographic analyses were of HPLC grade and purchased from Merck and VWR (Pennsylvania, United States).

### 2.2. In Vitro Digestion

Abiotic *in vitro* digestion was carried out according to Hollebeeck et al. in three stages (oral, gastric, and intestinal digestion) with 28 mg of affron® (equivalent to 2 tablets). Briefly, the salivary step runs for 5 min at pH 6.9 and 37°C, under aerobic conditions with 3.9 units *α*-amlylase/ml. Then, the samples were transferred for 90 min to the gastric step at pH 2 and 37°C with 71.2 units pepsin/ml under anaerobic conditions. Finally, the duodenal step was set for 150 min at pH 7 and 37°C, with 9.2 mg pancreatin/ml and 55.2 mg bile extract/ml under anaerobic conditions. Samples were taken from every steps and were centrifuged at 7000 g for 40 min at 4°C. The soluble fractions were frozen at −20°C, freeze-dried and the resulting dry powders were stored in a dark, dry place until analysis [30]. All samples were analysed at least in duplicate.

### 2.3. HPLC-PAD/MS Analyses of Saffron Extracts from the In Vitro Assay

Peak identification of affron® compounds was performed by mass spectrometry (MS) using an Agilent series 1100 HPLC, coupled to a mass-quadrupole detector (Hewlett-Packard, series 1100 MSD), with an electrospray ionization source (ESI) set at the following conditions: mass range, from 50 to 1500 amu; drying gas flow, 10 L/min; drying gas temperature, 340°C; nebulizer pressure, 40 psig; vaporizer temperature, 150°C; capillary tension, 2000 V; charge tension, 2000 V. Ion current acquisition was operated at both, positive and negative modes, with precision, accuracy, and detection limits as in [31].

High-performance liquid chromatography (HPLC) analysis of bioactive components from initial and digested saffron extract was performed using an Agilent Technologies 1220 Infinity series system equipped with a photodiode array detector (PAD) according to Kell et al. [12], while the analysis of crocetin was according to Umigai et al. [28]. The reference substances safranal, *trans*-crocin-4, kaempferol, and *p*-nitroaniline were used to plot external calibration curves for the quantification of safranal, crocetin-crocin isomers, kaempferol derivatives, and picrocrocin, respectively.

To estimate the percentage of bioaccessibility of the main bioactive compounds of affron®, an equation was used according to Ordoudi et al. [26].

### 2.4. Clinical Trial, Study Design, and Procedures

A single dose, randomized, double blinded study was used to evaluate the pharmacokinetics of two different saffron doses (56 mg and 84 mg). The randomization code was generated by Random Allocation Software, sealed envelope (London, UK). Enrolled participants were then allocated to one of two groups: group 1: consumed 4 × 14 mg of affron® tablets (56 mg single dose); group: 2 : 6 × 14 mg of affron® tablets (84 mg single dose).

This study was conducted in accordance with the ethical approval from Bellberry Limited ethics committee (approval number: 2016-04-305-A4). All participants provided written informed consent and were screened for inclusion and exclusion criteria prior to the conduct of the study.

The subjects of the pharmacokinetics were adult male (*n* = 5) and female (*n* = 8; nonpregnant and nonlactating) healthy volunteers of 18–30 years age. All participants self-reported to be in normal physical health (BMI < 30) and passed the exclusion screening. Exclusion criteria included suffering clinically significant medical condition, such as, but not limited to, cardiovascular, neurological, psychiatric, renal, immunological, endocrine (including uncontrolled diabetes or thyroid disease), or uncontrolled haematological abnormalities. Other exclusion criteria were the use within the past 3 months of the tested compounds and/or antioxidants; no current use of prescription medications except the oral contraceptive pill in women; and known allergy to any tested compound and/or antioxidant.

All participants were advised to fast from 10 : 00 pm prior to the study until the collection of the first blood sample. Fasting was broken immediately after dosing the corresponding tablets. At time 0 h and at 4 h after dosing, balanced breakfasts (410 kcal, approximately 30% of them from fats) and lunches (625 kcal, approximately 30% from fats) were served at the center. The meals were poor in carotenoids. Subjects remained onsite for the full first 6 hours of sample collection and returned to the center again for the 24 h samples. Subjects were monitored and asked to report any possible side-effects experienced due to intake of the tablets during the whole 24 h time after the intake of the tablets.

### 2.5. Analytical Procedure for Crocetin in Plasma

#### 2.5.1. Plasma Sample Collection

For crocetin pharmacokinetic analysis, blood samples (3 ml collected into tubes with EDTA) were drawn prior to affron® supplementation (time 0 h), between time 30 and 180 min at every 30 min and 24 h after supplementation. The blood tubes were briefly mixed by inversion and put on ice and centrifuged (600 g, 4°C for 10 min) to separate the plasma. The EDTA-plasma of every sample was carefully removed in 3 aliquots (500 *μ*l each) and stored at −20°C for less than 48 h before being finally transported and stored at −80°C.

#### 2.5.2. Sample Extraction

Plasma samples were extracted based on the method described and validated by Mohammadpour et al. [29]. Briefly, plasma samples were thawed to room temperature. Then, 20 *μ*l of an internal standard solution of 13-*cis*-retinoic acid and 120 *μ*l acetonitrile were added to 100 *μ*l of sample. The samples tubes were capped and vortexed for 10 s and put on ice for 5 min. The samples were then gently rotated for 10 minutes before centrifuging at 12000 g for 10 min. The supernatant was collected and analysed by HPLC.

#### 2.5.3. Recovery

The efficiency of the extraction process was estimated by the measure of the residual amount of the internal standard, and 13-*cis*-retinoic acid was recovered after the analysis. The recovery percentage was then applied to the results to calculate the true concentration of crocetin in the samples.

#### 2.5.4. Identification and Quantification of Crocetin in Plasma Samples

The chromatogram of a sample of pure standard crocetin and its typical absorption wavelength were used to identify and confirm the crocetin peak in every analysis, as shown in Mohammadpour et al. [29].

The crocetin concentration in biological samples was calculated using a standard linear curve at several crocetin concentration and the Shimadzu software for chromatogram analysis. All samples fell within the range of the linear standard curve (concentration (*μ*g/ml) = (AUC + 131.56)/9475). The intra-assay precision CV was 4.8%, and the interassay variability and precision CV were 7.3%, with a blood detection limit of 0.05 *μ*g/ml.

### 2.6. Pharmacokinetic Parameters and Statistical Analysis

Pharmacokinetic parameters were derived using GraphPad Prism 7.0 (California, USA). Data are represented as the mean ± SD and analysed using the SPSS 17.0 statistical software (SPSS Inc., Chicago, USA). Significance of differences at *p* ≤ 0.05 was determined by one-way ANOVA (Friedman test), and the Bonferroni test was used as post hoc analysis.

## 3. Results

### 3.1. Analytical Composition of the Saffron Extract

Individual identification of each component of the commercial saffron extract affron® was done by their characteristic UV absorbance peaks at 250, 310, and 440 nm, and their mass/charge relation, as in Table 1, shows the presence of the different bioactive components naturally present in saffron (*Crocus sativus* L.).

Among the main bioactive compounds found in affron®, at 250 nm, picrocrocin was detected, with characteristic quasimolecular (353.2 m/z) and fragment ions (151 m/z, 123 m/z) in ESI + mode. Kaempferol diglucoside was also identified at 250 nm, with its characteristic UV-Vis spectrum (maximum absorbance at 266 and 347 nm), together with typical quasimolecular ions in ESI+(633.2 *m/z*) and ESI− (609 *m/z*) modes. These results were in accordance with those obtained with saffron stigmas by Lech et al. [31]. Safranal was identified according to its retention time (identical to the standards) and UV-Vis spectra, with a characteristic maximum absorbance peak at 310 nm, as shown at Table 1.

With the previous method, crocetin detection was not possible due to interferences with crocin isomers. Residual crocetin was detected and identified by the identical retention time to the standard and its UV-Vis spectra, with a maximum absorbance peak at 440 nm.

### 3.2. In Vitro Digestion

As can be observed in Table 2, during the digestion process, the bioactive components of affron® were affected in a different manner. Referring to the volatile fraction, after the digestion process, safranal content significantly increased about two folds compared to the initial conditions (from 0.04 to 0.08%; Table 2), while the picrocrocin content decreased by 19%. This opposite behaviour is due to the mutual relation between both compounds. Safranal is formed by picrocrocin, and part of it may have been degraded during the digestive process, rising the safranal concentration. Compared with safranal, crocin isomers followed an opposite trend after the digestion process, which significantly reduced their concentration with a bioaccessibity of 40.77%. This great decrease in its content happened at the second stage; crocins were very sensitive to stomach conditions, *trans-*crocin-4 being the most sensitive crocin isomer to the acid-pepsin environment during gastric digestion (84% reduction.)

Furthermore, the whole *in vitro* digestion process (salivary, stomach, and duodenal steps) did not increase the concentration of crocetin compared to the initial composition of affron®, and its concentration remained 2 orders of magnitude lower compared with crocins. In addition, kaempferol derivatives content was also not affected during digestion, with high bioaccessibility data (69.23%; Table 2).

### 3.3. Pharmacokinetics with Humans

The demographic data of the subjects are shown in Table 3. All the adults included in the different study groups were young and in good shape, and none of the participants reported adverse events during the study.

**Figure 1:**
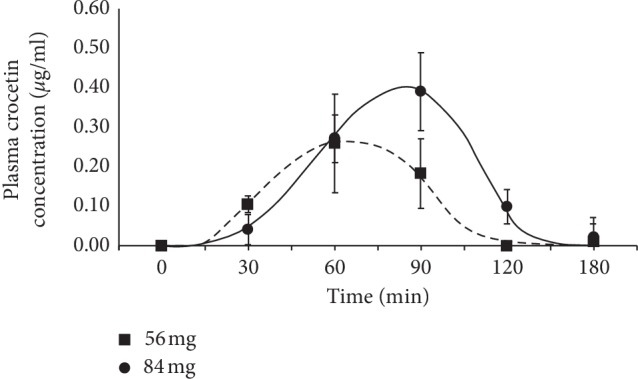
Concentration of crocetin in plasma after a single administration dose of affron®, the commercial saffron (*Crocus sativus* L.) extract of 4 tablets (56 mg) and 6 tablets (84 mg). The values represent mean ± SD.

Crocins, picrocrocin, and safranal were not detected in plasma. Only crocetin was enough concentrated to be identified and quantified at the tested blood samples (Table 4). The crocetin concentration in plasma up to 60 and 90 min, respectively, and then gradually decreased afterwards down to the HPLC-PAD quantification limit in 3 h (Figure 1). Twenty-four hours after affron® intake, the crocetin concentration remained below the detection limit.

Table 4 represents the differences in the pharmacokinetic parameters after the intake of the two different affron® dosages. At baseline, the two initial experimental groups showed low crocetin concentrations. Differences in AUC_(0-3h)_ and *C*_max_ between the two doses were statistically significant according to the Friedman test (*p* < 0.05). The average maximum concentration (*C*_max_) of crocetin was between 0.26 and 0.39 *μ*g/ml for the minimum (56 mg) and maximum (84 mg) dosages, obtained at 60 and 90 min, respectively, after oral intake (Figure 1).

## 4. Discussion

During *in vitro* digestion of affron® tablets, changes in the concentrations of the different bioactive compounds of interest could be observed. The increase of safranal content and decrease of picrocrocin concentration (Table 2) were because the picrocrocin hydrolysis to safranal occurred at moderate temperature and acidic pH during this *in vitro* assay [32,33]. On the other hand, kaempferol derivatives content did not decrease much during *in vitro* digestion. These results are in line with those obtained by Yang et al. [34], that described the changes during digestion of some kaempferol derivatives from a sample of kale, concluding that 69.4% of them were bioaccessible. Goh and Barlow also obtained similar results in their experiments, which showed a decrease of about 5% after digestion compared with the initial flavonoid content [35]. In addition, the significant of observed of crocin isomer concentration was probably due to the combination of different factors such as the physiological temperature (37°C) and pH changes at gastric and intestinal digestion phases, respectively [21]. The results obtained with the saffron extract tablets were similar to that by Kyriakoudi et al., showing a significant decrease of apocarotenoids, especially and more accelerated in the crocin *trans*-form. As a result of the extensive degradation, the crocin bioaccessibility after *in vitro* digestion was less than 50%. The experimental conditions were essentially as in Kyriakoudi et al. [21], except for stomach simulation duration, which was 50% longer in this work. Modifications suffered by crocins would happen quickly, and an extended period of treatment under acid conditions did not mean a further modification or alter much its content. In addition, the agreement between our results with a standardised saffron extract and those obtained with *in vitro* digestion of stigmas in a liquid formulation [21] suggest that neither the direct compression tablets manufacturing nor the tablet excipients did affect the quick solubilisation of crocins. During the *in vitro* digestion, the crocetin concentration compared with the initial composition of affron® present at the galenic formulation was not affected, proving that the formation of crocetin found in blood was not due to a simple chemical modification of the preexisting crocins but require the presence of enzymes from epithelial cells in the gastrointestinal tract, which hydrolyses the crocin isomers [22–24]. In order to study the appearance of crocetin in blood after oral consumption of an affron® tablet, the study was completed by means of crocin isomers pharmacokinetics in healthy volunteers.

Picrocrocin was absent in plasma, confirming its poor absorptivity [11]. Safranal was not detected or appeared below the detection limit in plasma, likely due to the very low amount of the substance in the tablet composition. Crocetin was detected instead of crocin isomers in blood, in agreement with the conclusions reported previously in animals [23, 24] and humans [27] confirming that a prior deglycosylation of crocin isomers is necessary before reaching the bloodstream. Nevertheless, none of previous works were carried out with a standardised saffron extract in such galenic form. The only one performed with saffron stigmas [27] with only 4 participants and unknown standardisation of the saffron infusion showed few remarkable results. Despite Chryssanthy et al. not aiming at doing a formal pharmacokinetics study, they found traces even after 24 h of administration, obtaining the highest crocetin concentration between 0.3 and 1.2 *μ*g/ml [27]. This was in a similar range to the *C*_max_ obtained in this study with an equivalent dose of 2 and 3 mg of crocins correspondingly.

There are two other works that studied the pharmacokinetics of crocetin. One of them used purified crocetin from dried fruits of *Gardenia jasminoides* [28], while the other used purified crocetin from saffron [29]. Crocetin doses in both cases were 10-fold higher than the used in this study. The results obtained by Umigai et al. with *G. jasminoides* crocetin was a *C*_max_ of 0.28 *μ*g/ml with a single oral dose of 22.5 mg of crocetin [28], whereas Mohammadpour et al. achieved a *C*_max_ of 0.35 *μ*g/ml with a single oral dose of 16 mg of saffron crocetins [29]. Surprisingly, those data are close to the *C*_max_ found in this work with tablets that contained only 2–3 mg crocins, giving evidence that crocetin derived from saffron extract crocins of a tablet was more bioavailable than the pure crocetin administration, perhaps because crocins are more bioavailable to enterocytes for later hydrolysis and absorption than the arrival of the crocetin itself.

The *T*_max_ obtained by crocetin after intake of the commercial saffron extract affron® tablet was just one h, suggesting that crocin isomers are metabolized in a short time such as some nutrients in healthy humans [36]. Surprisingly, the *C*_max_ values obtained in this study and in [29] with saffron were found much earlier than those obtained with *G. jasminoides* in [28]. The delayed bioavailability found in gardenia crocetin may be explained by the fact that the burden of crocetin derivates isomers in saffron and gardenia is different. While most of the saffron crocins are found in *trans*-form as seen in Table 1, some of those from *G. jasminoides* are found in *cis*-form [37] with specific isomers not found in saffron [38]. Differential bioavailability has been described in isomers of the same structural carotenoids in humans, such as in lycopene [39]. Remarkably, the obtained *T*_max_ value found in our work is considered much lower than that obtained from pharmacokinetic studies of other C40 carotenoids of interest in health such as zeaxanthin, lutein, lycopene, or *β*-carotene, where several hours are required to reach the plasma maximum concentration [39–43], showing that crocetin presented in this galenic form is quickly absorbed than the other carotenoids.

The distribution volume obtained in this work was 3.18 L, which is related to compounds that bind strongly with plasma proteins as albumin [44], reducing its free content in blood. It correlates with the previous works which described that crocetin binds to albumin [45, 46] and other human serum proteins [47]. After 3 h of administration, crocetin ceased to be detected, obtaining a plasmatic half-life of 0.85 ± 0.04 h and a clearance of 2.70 ± 0.14 L/h. The obtained values suggest that the half-life of crocetin was very short, and its free form in blood was no longer detected, not in agreement with the previously observed results that could be detected 24 h after consuming much higher amounts of crocins [27]. This may be because, as mentioned, its distribution volume suggests that it binds strongly to albumin, leaving the free levels below of its detection limit of 0.05 *μ*g/ml [29].

The fast presence of crocetin in blood plasma after the administration of the saffron extract contributes to better explain saffron beneficial effects in animal models [48]. The commercial standardised saffron extract affron® used in this study is related to beneficial effects in depression and mood disorders by several clinical trials at the dosage used in the present work [13, 14, 16] and in the reduction of inflammatory damage in the retina in an animal model of glaucoma [20]. The obtained results in this study increase the interest of saffron extract use as a new preventive therapy that requires a fast-active countereffect, for instance, as “fast-track” carotenoid in the Aged-Related Eye Disease Study (AREDS-2) formula.

## 5. Conclusion

Even though crocin isomers bioaccessibility were approximately 40% after affron® tablet digestion, they are able to reach the bloodstream, with proven functional effect, in crocetin form more quickly than other lipophilic carotenoids of higher-molecular mass, being an interesting alternative for use as in fast-acting formulations. Besides, with a low dose of crocin from affron® tablet (2–3 mg), the obtained values of *C*_max_ of crocetin were similar to the values obtained from other studies with higher doses of purified crocetin. In brief, affron® galenic presentation showed rapid absorption and high oral bioavailability.

## Figures and Tables

**Table 1:** Chromatographic identification of the main bioactive components found in affron® by HPLC coupled to the mass detector in ESI+ and ESI−.

RT (minutes)	Compound	Maximum UV-Vis (nm)	ESI+ (m/z)	ESI− (m/z)
7.6	Picrocrocin	251	511.2; 365.1; **353.2**; 337.1; 329.1; 185.1; 159.9; **151.1**; **123.0**; 81.3	1480.8; 667.2; 517.1; 385.0; 181.2; 153.1
11.5	Kaempferol diglucoside	266; 347	**633.2**; 347.1	**609.0**; 284.0
22.1	*trans*-crocin-4	261; 323; 441; 464	**999.4**; **675.2**; **511.2**; 347.1	1336.2; **651.2**; 326.8; 283.1
26.7	*trans*-crocin-3	261; 323; 440; 463	**837.4**	
30.7	*trans*-crocin-2′	261; 328; 440; 464	**675.2**; 593.0; **513.2**; 351.1; 130.1; 102.2; 74.1	
33.9	Safranal	313	—	—
36.2	*cis*-crocin-4	225; 262; 325; 433; 456	**999.4**; 821.3; 668.3; 611.0; 391.0; 347.0	**651.2**
37.6	*trans*-crocin-2	260; 322; 433; 458	**675.2**; 541.1	**651.2**; 327.1; 311.1; 283.1

Bold numbers indicate coincidences in the molecular fractions obtained in [30].

**Table 2:** Bioactive molecules content expressed as percentage (%, dry basis) found in affron® before and after *in vitro* digestion process at salivary, gastric, and duodenal steps.

Bioactive components	Initial sample	Digested sample			Bioaccessibility
		Salivary	Gastric	Duodenal	
Safranal	0.04 ± 0.01^a^	0.03 ± 0.00^a^	0.21 ± 0.01^c^	0.08 ± 0.01^b^	200.00
*t*-crocin-4	1.66 ± 0.04^b^	1.11 ± 0.43^b^	0.26 ± 0.03^a^	0.20 ± 0.04^a^	12.05
Total crocins	3.63 ± 0.05^b^	3.43 ± 0.12^b^	1.87 ± 0.35^a^	1.48 ± 0.22^a^	40.77
Kaempferol diglucoside	0.13 ± 0.01^b,c^	0.14 ± 0.01^c^	0.12 ± 0.01^b^	0.09 ± 0.01^a^	69.23
Picrocrocin	3.21 ± 0.07^c^	3.14 ± 0.11^c^	2.07 ± 0.02^a^	2.61 ± 0.05^b^	81.31
Crocetin	0.03 ± 0.01^a^	0.02 ± 0.01^a^	0.03 ± 0.01^a^	0.02 ± 0.01^a^	66.67

Data are represented as mean ± SD. Different letters within a column (a–c) indicate significant differences (*p* < 0.05) of the bioactive component of affron® in every step during the digestion process.

**Table 3:** Baseline demographic details of participants.

Demographics	Total (*N* = 13)	Treatment groups	
		affron® 56 mg (*n* = 7)	affron® 84 mg (*n* = 6)
Mean age (SD)	23.6 (2.9)	23.0 (2.0)	24.6 (3.9)
Age range (years)	19–30	20–26	19–30
Gender (number, %)			
Female	8 (61.5%)	4 (57.1%)	4 (66.6%)
Male	5 (38.5%)	3 (42.9%)	2 (33.4%)
Weight (SD)			
Female	59.0 (4.2)	58.9 (3.7)	59.1 (4.8)
Male	83.2 (5.7)	82.9 (5.3)	83.6 (6.1)

**Table 4:** Pharmacokinetic parameters of crocetin plus derivatives that come from the metabolism of crocin isomers.

Parameters	Treatment groups	
	affron® 56 mg	affron® 84 mg
*T*_max_ (min)	60	90
*C*_max_ (*μ*g/ml)	0.26 ± 0.12	0.39 ± 0.10
Total AUC_(0-3h)_ (*μ*g·h/ml)	21.07	26.15
*T*_1/2_ (h)	0.85 ± 0.04	
CL (L/h)	2.70 ± 0.14	
Vd (L)	3.19 ± 0.00	

Total AUC is calculated on the crocetin concentration change from baseline data. *k*_e_, *T*_1/2_, CL, and Vd are represented as the mean ± SD. *T*_max_ = time to maximum concentration; *C*_max_ = maximum concentration; AUC = area under the curve; *T*_1/2_ = half-life time; CL  =  clearance; Vd = distribution volume.

## Data Availability

The data used to support the findings of this study are included within the article.
